# Improving translational studies: lessons from rare neuromuscular diseases

**DOI:** 10.1242/dmm.022616

**Published:** 2015-10-01

**Authors:** Dominic Wells

**Affiliations:** Department of Comparative Biomedical Sciences, Royal Veterinary College, London, NW1 0TU, UK

## Abstract

Animal models play a key role in the development of novel treatments for human disease. This is particularly true for rare diseases – defined as disorders that affect less than 1 in 2000 people in the human population – for which, very often, there are no effective methods of treatment. Pharmaceutical companies are increasingly focussing on the development of therapies for the more than 7000 rare diseases. Because the majority of these are the result of single gene disorders, the exceptional ability to manipulate the mouse genome means that many such studies will take place in the laboratory mouse. But how good are the mouse models and how useful are they in assessing the potential for translational medicine? In this Editorial, I will discuss current difficulties in translational research as well as examples of good laboratory practice and guidelines that are being implemented to improve the translational potential of animal studies in the field of neuromuscular rare diseases. This could represent a potentially useful approach for adoption by other disease fields to achieve a greater success rate in translational studies.

## Abstract (precis)

**Drug Discovery Collection:** This Editorial reviews problems and some solutions to the translational use of animal models in the rare neuromuscular diseases context. This can serve as a model for other disease fields.

## Lost in translation: improving animal experiments

Despite continuous research efforts to advance our understanding of rare diseases and to discover potential targets for therapy, 20 years ago there was little interest in rare diseases from pharmaceutical companies. However, changes in the regulatory pathways, a decrease in the space for development of blockbuster drugs (drugs that generate annual sales of at least $1 billion), increased societal criticism of the pharmaceutical industry, and cost models pioneered by companies like Genzyme have made rare diseases both a potentially profitable investment and a public relations winner. The rare diseases space allows for the development and approval of novel technologies and targets that could go on to have very wide applications. For example, inhibition of myostatin, a negative regulator of muscle mass, has the potential to not only address some of the problems associated with a number of neuromuscular disorders but might also be applicable to the treatment of muscle wasting associated with bed rest for a variety of medical conditions as well as the loss of muscle mass and function associated with aging (Jasuja and LeBrasseur, 2014). This would indeed be a blockbuster drug but with the proof-of-principle and initial registration via rare diseases.

There are concerns that animal models only provide limited and potentially misleading results that could negatively impact subsequent human clinical trials. Indeed, there are many examples of animal experiments that have preceded unsuccessful clinical trials, such that critics have argued that animal experiments are misleading or even of no value. The alternative view is that animal experiments are informative in the majority of cases but only if we conduct them appropriately and analyse them critically with care. So, what are the major problems with many animal experiments to date?

“…critics have argued that animal experiments are misleading or even of no value. The alternative view is that animal experiments are informative in the majority of cases but only if we conduct them appropriately and analyse them critically with care”

Firstly, and perhaps most importantly, animal experiments to develop new therapies need to be conducted with translation in mind. It is vital that such studies should use doses that can realistically be applied to humans, with appropriate metabolic scaling calculations, as opposed to dosing to a maximum effect that might not be possible in human studies where safety is of over-riding importance. Rodents are remarkably resilient: mice can survive a doubling of their blood volume in 10 seconds, an event that would be lethal to larger animals. Indeed, as a prey species, mice show very few signs of discomfort, which might mislead investigators into thinking that high doses of a drug have no adverse effects. Routes of administration that are commonly used in rodents, such as intraperitoneal injections, will not be used in humans and may result in very different pharmacokinetics.

It is also essential that the results of treatments are evaluated in an appropriate manner that reflects the translational relevance of the animal model and is not potentially subject to other interpretations. Outcome measures used in testing the effects of a potential therapy in an animal model should be carefully considered. Examples of these problems are given below.

In addition, it is vital that the animal experiments are conducted using good laboratory practice and are reported in full; for example, in compliance with the ARRIVE guidelines (Kilkenny et al., 2010). Randomisation and blinding to treatment are absolutely essential in order to give confidence that the analysis has been performed without unintentional bias. Replication in another independent laboratory is also a very important safeguard to ensure that clinical development is not based on an erroneous set of results. If the group developing the novel therapy is concerned about intellectual property (IP), this should not be a barrier: the confirmatory studies can always be undertaken using a contract research organisation that makes no demands on IP but merely acts on a fee-for-services basis.

## Learning from animal models of neuromuscular diseases

An examination of several animal models for neuromuscular disease might help to illustrate many of the above issues.

Duchenne muscular dystrophy (DMD) is an X-linked lethal muscle wasting disorder characterised by repeated degeneration of skeletal muscle, initially followed by regeneration but this fails over time as muscles become fibrosed and fat replaces the lost muscle (Bushby et al., 2010; Kinali et al., 2011). The disease is due to mutations in a very large gene, the *DMD* gene, encoding the large protein dystrophin, which links the internal cytoskeleton of the muscle fibre with the extracellular matrix. The condition affects about 1 in 5000 live male births and has a similar incidence regardless of race or geographical location (Mah et al., 2014). The *mdx* mouse is the most commonly used model of DMD and arose from a spontaneous murine *Dmd* mutation in a colony of C57BL/10 mice (Bulfield et al., 1984). Many of the more than 2500 papers published using the *mdx* mouse have evaluated a wide range of potential treatments, but relatively few have been translated to successful clinical trials. Many of the studies have involved treatment of young *mdx* mice, often starting at 4-6 weeks old. However, at this age the mouse is still developing the disease, having shown no histopathological evidence of disease until 3 weeks of age followed by a sudden onset of muscle necrosis that eventually involves most of the skeletal muscle fibres and then stabilises to a less florid pattern of necrosis by 8-10 weeks of age (Grounds et al., 2008). In contrast, extensive muscle damage can be detected in neonates with DMD and, for the foreseeable future, all DMD patients will have established disease at the time treatment is initiated. Thus, data from short-term treatment of *mdx* mice with developing disease might not have significant translational value for DMD patients. A second problem is that the dose reported for many of the *mdx* studies is simply impossible to achieve in man without severe side effects. A third problem is that very few of the published manuscripts make any mention of randomisation and blinding to avoid bias. Finally, a fourth problem is that many of the measures used to determine the effect of treatment are assessed differently from lab to lab, making comparison of different treatments effectively impossible.

The muscular dystrophy community has tried to address this final problem by publishing standard operating procedures for many of the common methods that have been validated by a panel of experts. This consensus approach was under the aegis of Treat-NMD, an international consortium of clinicians and scientists dealing with neuromuscular diseases originally established as an EU-funded ‘network of excellence’ that launched in January 2007. The standard operating procedures can be accessed through the Treat-NMD website (http://www.treat-nmd.eu/research/preclinical/dmd-sops/). Even so, some of the measures can have confounding influences. One of the often-raised criticisms of the *mdx* model is that, unlike DMD patients, who lose independent ambulation by 12 years old and without medical intervention die by 16 years old, the mice show no easily recognisable clinical signs of muscular dystrophy and only have a moderately shorter lifespan than wild-type mice (Chamberlain et al., 2007). Attempts have been made to develop whole-body tests of muscle strength and behavioural monitoring to capture the effects of muscular dystrophy in the *mdx* mice, and these have been used to evaluate treatment effects. However, such tests might be confounded by drugs that modify CNS processes because these might change the motivational state of the mice, either obscuring beneficial effects of treatment or giving a falsely positive impression of these effects.

However, there are a number of assays that show the similarities of the disease in mouse and man and that can be used to more accurately reflect the translational potential of novel treatments. Histopathology in the *mdx* mouse is similar to the early stage of DMD, although the limb muscles do not develop the substantial fibrosis and fatty infiltration seen in older DMD muscle. The diaphragm is an exception because it develops a very marked fibrosis and associated fibre loss (Stedman et al., 1991). Drugs that decrease the progression of fibrosis in the *mdx* diaphragm are quite likely to have utility in DMD. Muscle physiology in the *mdx* mouse also correlates well with the human condition. Dystrophic muscle in both mouse and man is highly vulnerable to damage as the result of eccentric exercise (lengthening contractions), and drugs that protect the muscle against eccentric damage are also likely to translate well. Finally, the maximum specific twitch and tetanic forces are reduced in dystrophic muscle and can be improved by some drug treatments or the restoration of dystrophin (e.g. Godfrey et al., 2015). An important point that is often overlooked is the extent to which the therapeutic intervention in the *mdx* mouse restores the measure to that of the wild-type mouse. A useful evaluation is the concept of the recovery score developed by Jean-Marie Gillis in the context of analysing transgenic dystrophic mice (Gillis, 2002), where the percentage change in any measure from the *mdx* to the wild-type indicates the level of the treatment effect.

Amyotrophic lateral sclerosis (ALS) is another muscle-wasting disease but, in contrast to DMD, ALS arises from the loss of the motoneurons innervating the muscles. Diagnosis is commonly about the age of 50 and survival after diagnosis is generally only 2-4 years. About 10% of cases have a familial basis, whereas the remaining 90% seem to be sporadic. Of the familial cases, about 20% have been linked to gain-of-function mutations in the Cu/Zn superoxide dismutase (*SOD1*) gene (Ingre et al., 2015). The most commonly used animal model for ALS is the high-copy-number SOD-1 G93A transgenic mouse, which develops hindlimb muscle wasting and paralysis by 120-130 days old. More than 1000 papers have been published on this model, many describing successful prolongation of survival with a range of drugs. Only one of these – Riluzole – has successfully completed clinical evaluation and on average only offers a few extra months of survival (Miller et al., 2012). So why has translation proved so difficult in these studies? An excellent detailed analysis has been provided by Scott and colleagues (2008) in which they identified a number of common flaws in the studies, including inadequate numbers, differences in males and females (many reports failing to reveal the sex of the mice used), litter-to-litter variation, failure to censor non-ALS related deaths, variation in copy number of the transgene, and the use of inappropriate statistics. They outlined a robust experimental approach to avoid these problems but, regrettably, relatively few of the studies published in the last 6 years have conformed to these guidelines.

## How to improve translation to clinical trials

Another problem with the development of therapies for rare diseases is the target population. In many rare diseases the natural history of patients is not well documented or is changing as a result of improved medical management, which makes it difficult to develop outcome measures that show the clinical benefit of treatment. Furthermore, the rarity of the disease often means that clinical trials need to involve multiple clinical centres and often require multinational collaborations. Pharmaceutical and biotechnology organisations often do not have the in-house expertise in the rare disease and a good understanding of the reality of clinical trials in such populations. For novel therapies, the majority of ideas and initial experiments are performed by academics and clinicians but, in many cases, these individuals lack experience of how to take such ideas to clinical trials and the route to drug approval. The neuromuscular community recognised these issues and, in 2009, the Treat-NMD international neuromuscular collaboration set up the Treat-NMD Advisory Committee on Therapeutics (TACT) to provide advice on drug development for neuromuscular diseases. Experience from TACT reviews of drug development programmes has shown the importance of performing high-quality studies in animal models to underpin drug development proposals (Heslop et al., 2015).

“For novel therapies, the majority of ideas and initial experiments are performed by academics and clinicians but, in many cases, these individuals lack experience of how to take such ideas to clinical trials and the route to drug approval”

It is important to recognise that a statistically significant improvement in some measure in an animal model might not be biologically important and so will lack real translational predictivity. As a generality, a relatively small change in an animal model is a poor basis for the development of a therapeutic programme, whereas large improvements in multiple measures provide more confidence. The very strong desire to develop treatments for lethal inherited conditions such as DMD, often coupled with an enthusiasm for a particular technology or class of drugs, can lead to wishful thinking and a failure to critically evaluate the results of studies in animal models of the disease, and this needs to be avoided if we are to see a greater success rate in translational studies.

“As a generality, a relatively small change in an animal model is a poor basis for the development of a therapeutic programme, whereas large improvements in multiple measures provide more confidence”

## Conclusions

A careful consideration of the drug dose, route of delivery and life history of the human disease are essential elements in designing better translational studies in mouse models. An understanding of the similarities and differences between the mouse model and the human disease is vital in defining the most appropriate outcome measures for the animal experiments. The development of standard operating procedures for such outcome measures allows improved independent validation of the results. Finally, a critical evaluation of the treatment effect, rather than just a statistical improvement, should be used to inform future clinical development. Combining all of these elements should increase the success rate and minimise wastage in the development of new treatments for disease.
